# Carboxymethyl chitosan stabilized AuNPs/ACP nanohybrids in enamel white spot lesions

**DOI:** 10.3389/fbioe.2024.1421887

**Published:** 2024-07-16

**Authors:** Xiaohua Chen, Hengyu Liu, Qianqian Zhang, Xuehua Chen, Lihui Wang, Yanling Yu, Yuanping Hao

**Affiliations:** ^1^ School of Stomatology of Binzhou Medical University, Yantai, China; ^2^ Qingdao Stomatological Hospital Affiliated to Qingdao University, Qingdao, China; ^3^ Yantai Central Blood Station, Yantai, China; ^4^ Department of Stomatology, School of Shandong Second Medical University, Weifang, China

**Keywords:** carboxymethyl chitosan, gold nanoparticles, amorphous calcium phosphate, antimicrobial, remineralization, white spot lesions

## Abstract

Acidic bacterial biofilms-associated enamel white spot lesions (WSLs) are one of the hallmarks of early caries, causing demineralization and decomposition of dental hard tissues. Therefore, to effectively prevent and treat WSLs, it is important to inhibit the activity of cariogenic bacteria while promoting the remineralization of demineralized enamel. Amorphous calcium phosphate (ACP) favors hard tissue remineralization due to its biological activity and ability to release large amounts of Ca^2+^ and PO_4_^3-^. However, ACP-based biomineralization technology is not effective due to its lack of antimicrobial properties. Here, carboxymethyl chitosan (CMCS) was employed as a reducing agent and stabilizer, and dual-functional nanohybrids CMCS/AuNPs/ACP with biofilm resistance and mineralization properties were successfully synthesized. The addition of AuNPs enhances the antimicrobial activity and participates in regulating the formation of hydroxyapatite (HAp). The nanohybrids exhibited significant destructive effects against cariogenic bacteria and their biofilms and showed bactericidal activity under bacteria-induced acidic conditions. More importantly, this nanohybrids showed superior results in promoting the remineralization of demineralized enamel, compared to fluoride and CMCS/ACP *in vitro*. The CMCS/AuNPs/ACP nanohybrids not only reverse the cariogenic microenvironment at the microbial level, but also promote self-repairing of enamel WSLs regarding the microstructure. The present work offers a theoretical and experimental basis for using the CMCS/AuNPs/ACP nanohybrids as a potential dual-functional agent for the clinical treatment of enamel WSLs.

## 1 Introduction

Dental caries is the localized damage to the sensitive hard tissues of teeth caused by acidic byproducts from bacterial fermentation of dietary carbohydrates (Kidd and Fejerskov, 2003; Sims et al., 2020). With the progress of human beings and the development of social economy, our food has become increasingly fine, and fine food tends to adhere to the surface of tooth enamel, which becomes a natural medium for caries-causing bacteria (largely *Streptococcus mutans*) (Bhadila et al., 2020). Endogenous bacteria in the tooth-surface plaque biofilms produce weak organic acids as a byproduct of fermentable carbohydrate metabolism (Caufield and Griffen, 2000; Fejerskov, 2004; Scheie and Petersen, 2004). That acid leads to localized pH values below a critical value, resulting in an imbalance between demineralization and remineralization of the enamel, then resulting in demineralization of tooth tissues (Caufield and Griffen, 2000; Featherstone, 2000; Featherstone, 2004). Demineralization can be reversed at an early stage by the uptake of calcium, phosphate, and fluoride (Selwitz et al., 2007). As a sign of early dental caries, enamel white spot lesions (WSLs) refer to the demineralized enamel, characterized by opaque, matte, and chalky enamel without enamel defects (Chang et al., 1997; Chapman et al., 2010; Feldens et al., 2016). If left untreated, with gradual loss of minerals, WSLs can develop into true dental caries, especially in people at high risk for dental caries (Sudjalim et al., 2006).

In order to stop caries from occurring, it is critically important to treat WSLs early, therefore we have to break the imbalance between demineralization and remineralization, then remineralization of the enamel occurs. Fluoride is our most commonly used remineralizing agent, which effectively inhibits enamel demineralization and enhances remineralization (Wiegand et al., 2007). However, the remineralizing effect of fluoride is limited by salivary Ca^2+^ and PO_4_^3-^ levels (Lu et al., 2023). Moreover, in terms of resistance to bacterial biofilms, it has been found that fluoride has little or no sustained effect on cariogenic biofilm cells even at high concentrations (Dang et al., 2016). Another issue that cannot be ignored is the cytotoxicity of fluoride ions (Zuo et al., 2018). Fluoride is found in drinking water and a variety of oral hygiene products, and there are potential health risks if excessive fluoride is ingested (Burt, 1992; Browne et al., 2005). It has been found that remineralization can be achieved by exposing demineralized enamel to solutions containing Ca^2+^ and PO_4_^3-^ (Moreno and Zahradnik, 1979). The biomimetic strategy for enamel remineralization, based on the natural process of enamel crystallization (Cölfen and Mann, 2003), has been widely studied for its biomimetic mineralization ability (Chen et al., 2015; Wang et al., 2017).

Amorphous calcium phosphate (ACP) is an excellent biomimetic remineralizing agent with good biological activity and releases large amounts of Ca^2+^ and PO_4_^3-^ (Kim et al., 2010; DeRocher et al., 2020). However, ACP is an unstable substance and will lose its remineralization properties if it crystallizes prematurely as hydroxyapatite (HAp) in aqueous solution (He et al., 2022). Amelogenin, casein phosphopeptides, chitosan derivatives, poly-amidoamine, polyelectrolytes, and poly (carboxybetaine acrylamide) can stabilize Ca^2+^ and PO_4_^3-^ to form ACP through tyrosine-rich fragments, primary reactive sequences, amino groups, and carboxyl groups, respectively, and their remineralization capacity has been proven (He et al., 2022; Yan et al., 2022). Natural polysaccharides such as chitosan (CS) have received extensive attention in biomedicine (Jiang et al., 2022). As a hydrosoluble derivative of CS, carboxymethyl chitosan (CMCS) has become the focus of attention in many biomedical fields due to its remarkable antimicrobial properties, biodegradability, biocompatibility and non-toxicity (Patrulea et al., 2015; Shariatinia, 2018). CMCS as a carboxyl-rich amphoteric electrolyte, can stabilize ACP under neutral (negatively charged) or acidic (positively charged) conditions (He et al., 2019; Lin et al., 2019). The study proved that CMCS/ACP nanocomplexes can promote remineralization of dental tissues (Chen et al., 2015; Xiao et al., 2017), however, the inhibitory effect of CMCS/ACP on cariogenic biofilm was not satisfactory (He et al., 2019).

In addition to providing the essential elements for the formation of HAp, the achievability of enamel remineralization also needs to be based on plaque control; if plaque biofilm is not controlled, it will continue to produce an acidic environment, leading to continued demineralization of enamel; therefore, antimicrobial plaque biofilm is particularly important in remineralization of dental tissues (Melo et al., 2013; Simeonov et al., 2019). Nanoparticles, with their large specific surface area and high charge density, are able to better contact and act when interacting with negatively charged bacterial cell surfaces, thus enhancing antibacterial activity (Cao et al., 2018). Among them, gold nanoparticles (AuNPs) have attracted our attention due to their good biocompatibility (Lima et al., 2019), their application in enhancing the antimicrobial activity of materials (Xie et al., 2020), and their ability to induce the formation of HAp by transporting calcium ions (Sari et al., 2022). AuNPs are characterized by their small size and large surface area, and their antimicrobial activity does not induce any ROS-related processes (Cui et al., 2012; Bindhu and Umadevi, 2014). AuNPs have received safety approval by the U.S. Food and Drug Administration (FDA) for use as antimicrobial drugs because of their excellent biocompatibility for mammalian cells (Yang et al., 2019). Additionally, acidic proteins or macromolecules with negatively charged groups control most of the biomineralization processes, and these macromolecules are thought to control crystal nucleation, polymorphism, and growth (Rautaray et al., 2005). In particular, AuNPs stabilized by amino acids can serve as templates for HAp growth and promote the formation of HAp (Lee et al., 2002; Sari et al., 2022).

In previous studies, members of our group have demonstrated that ACP effectively promotes remineralization of demineralized enamel (Lu et al., 2023). Therefore, in this study, a novel ACP-based nanohybrids with antimicrobial and remineralization capabilities was developed to counteract enamel WSLs using biomineralization technology. The objectives of this study were to: (1) develop a bioactive nanohybrids with enhanced antimicrobial and remineralization properties using CMCS as a reducing agent and stabilizer for the first time, and (2) evaluate antibacterial and anti-biofilm effects of nanohybrids and remineralization in a demineralized enamel model. So far, there are no reports on CMCS-stabilized AuNPs/ACP-conjugated nanohybrids in inhibiting WSLs and preserving the structural minerals of teeth.

## 2 Experimental section

### 2.1 Materials

CMCS (BR) and Calcium chloride dihydrate (CaCl_2_.2H_2_O) were obtained from Macklin Reagent Co., Ltd. (Shanghai, China). Chloroauric acid (HAuCl_4_) was provided by Shanghai Yuanye Bio-Technology Co., Ltd. (Shanghai, China). Dipotassium hydrogen phosphate (K_2_HPO_4_) was purchased from Aladdin Reagent Co., Ltd. (Shanghai, China). All remaining chemicals and solvents were analytical reagents and employed without further purification upon receipt.

### 2.2 Preparation of CMCS/AuNPs/ACP nanohybrids

i) 2.2% (w/v) of CMCS solution in the reaction vessel was mixed with 6 mL of 0.25 mol/L NaOH in a thermostatic magnetic field, heated to 70°C and then 6 mL of 0.38% (w/v) HAuCI_4_ solution was added. CMCS/AuNPs mixture was obtained after 20 min at 70°C, and then the solution was adjusted to neutral with 1 M HCl solution.
ii) 41.76 mg K_2_HPO_4_ was dissolved in 10 mL water with stirring under 500 rpm.
iii) 58.8 mg CaCl_2_.2H_2_O was added into 30 mL CMCS/AuNPs mixture at 500 rpm until the CaCl_2_.2H_2_O was completely dissolved. Subsequently, the K_2_HPO_4_ solution was slowly dripped into it, and then continuously stirred gently for 20 min to form CMCS/AuNPs/ACP nanohybrids.

### 2.3 Characterization

The UV-Vis absorption spectrum of CMCS/AuNPs was recorded by a UV-8000 UV-Vis spectrophotometer (Yuanxi, China). The size and shape of CMCS/AuNPs/ACP nanohybrids were examined by transmission electron microscopy (TEM, JEM-2100UHR, JEOL, Japan). The particle size distribution was measured by ImageJ software. A Nicolet iN10 Fourier transform infrared (FTIR) spectrometer (Thermo FisherScientific, Waltham, MA, United States) was employed to analyze the FTIR spectra of CMCS, CMCS/AuNPs, and CMCS/AuNPs/ACP, which was performed with an attenuated total reflectance-infrared (ATR-IR) system in the range of 500–4,000 cm^-1^. The surface zeta potentials of CMCS/AuNPs and CMCS/AuNPs/ACP were measured using the Zetasizer Nano ZS (Malvern instrument, United Kingdom).

### 2.4 Preparation of tooth enamel samples

The third molar was obtained from 18 to 30-year-old patients who underwent wisdom tooth extraction in Qingdao Stomatological Hospital, China. The study content was approved by the Ethics Committee of Qingdao Stomatological Hospital (2022KQYX024). Inclusion criteria were mature teeth without caries, cracks, or other defects. Clean the tooth surfaces under flowing deionized water (DW). Afterward, the tooth crowns were removed from their roots by cutting with a low-speed diamond saw that is water-cooled. The enamel samples were obtained after cutting the tooth crowns. The enamel samples were polished with SiC papers (5,000 particle size) under flowing deionized water. Then the samples were stored in 1% thymol solution at 4°C before use.

### 2.5 *In vitro* antibacterial and antibiofilm properties of CMCS/AuNPs/ACP nanohybrids

#### 2.5.1 Antibacterial properties of the CMCS/AuNPs/ACP nanohybrids

*S. mutans* is a gram-staining positive coccus, which is one of the largest species within the genus *Streptococcus* in oral natural flora and is one of the most common caries-causing bacteria. Using *S. mutans* (BNCC 336931), *Escherichia coli* (BNCC 133264), and *S. aureus* (BNCC 186335) as experimental subjects, the culture medium of *S. mutans* was Brain Heart Infusion (BHI) medium (Solarbio, China), *E. coli* was LB Broth medium (Solarbio, China), and *S. aureus* was Tryptone Soy Broth (TSB) medium (Solarbio, China), all of which were cultured under routine aerobic environment at 37°C in a constant temperature incubator (SPX-150, SaiFu, China). We evaluated the antibacterial properties of CMCS, CMCS/AuNPs and CMCS/AuNPs/ACP *in vitro* by colony-forming units (CFU) method. After co-culture with materials at 37°C for 24 h, bacteria were titrated on agar plates by gradient dilution. We used a digital camera to record the CFUs on the agar plates. Each group was repeated 3 times and the number of viable colonies in each group was calculated and the results were described as bactericidal rate:

$$\text{Bactericidal rate}=\frac{{\text{CFU}}_{0}-{\text{CFU}}_{1}}{{\text{CFU}}_{0}}\times 100\%$$

Where ${\text{CFU}}_{0}$ and ${\text{CFU}}_{1}$ are the number of colonies in the control and experimental groups, respectively.

To observe the morphology of bacteria, *S. mutans*, *E. coli*, and *S. aureus* were co-cultured with CMCS, CMCS/AuNPs, and CMCS/AuNPs/ACP for 24 h, then washed with sterile Phosphate buffers (PBS), fixed with glutaraldehyde, centrifuged and then adsorbed by carbon-supported membranes. Fixation bacteria were observed by transmission electron microscopy (TEM, JEM-2100UHR, JEOL, Japan).

#### 2.5.2 Antibiofilm properties of the CMCS/AuNPs/ACP nanohybrids

Non-irritating saliva samples were collected from six healthy adult volunteers, mixed, and centrifuged at 4,000 *g* at 4°C for 20 min. Then the supernatant was obtained by a 0.22-micron filter (SLGPR33RB, Millipore, China) to remove bacteria. The enamel samples were washed with PBS and subjected to a 30-min saliva immersion at 37°C for the acquisition of salivary-acquired pellicles. The saliva-coated enamel samples were placed on a 24-well plate, and 1,000 µL diluted bacterial suspension (1 × 10^8^ CFU/mL) were co-cultured with these samples at 37°C for 48 h to form the biofilm. The medium was changed every 24 h. Biofilms were gently rinsed with PBS for three times after 48 h. Then the CMCS/AuNPs/ACP nanohybrids (2.1 μg/mL, 200 µL) were carefully added to each group of corresponding wells and incubated at 37°C for 24 h. Biofilms were gently rinsed with PBS for three times and stained using the LIVE/DEAD™BacLight™ Bacterial viability kit (Solarbio, China) according to the instructions. Confocal microscopic imaging of stained bacteria was performed on a Confocal laser scanning microscope (CLSM, SP8, Leica Microsystems). NucGreen and EthD-III are nucleic acid dyes. When NucGreen and EthD-III are used together, bacteria with intact cell membranes show green color, while bacteria with damaged cell membranes show green color and red color under different channels, respectively.

Biofilm biomass was then detected by the crystal violet (CV) staining. The bacterial suspension (1 × 10^8^ CFU/mL) was inoculated into a 24-well plate and incubated in an incubator at 37°C. The medium was changed every 24 h. Then the CMCS/AuNPs/ACP nanohybrids (2.1 μg/mL, 200 µL) were carefully added to each group of corresponding wells and incubated at 37°C for 24 h. Biofilms were gently rinsed three times with sterile PBS, then stained with 0.1% CV staining solution (1 mL) for 20 min. After sucking up the CV dye and gently washing with sterile PBS (2 mL), the biofilm was solubilized with anhydrous ethanol (1 mL). The biomass of the biofilm was quantified by recording the absorbance at nm.

### 2.6 Measurement of enamel remineralization *in vitro*

The enamel samples were repeatedly wiped with anhydrous ethanol, flushed with mass DW, and then ultrasonic washed in DW for 30 min. Finally, the *ex vivo* teeth demineralization models were fabricated by etching with 37% phosphate gel acid for 30 s. The acidulated area of each enamel sample is 4 × 3 mm^2^, the rest was covered with two coats of acid-resistant nail polish. The demineralized dental samples were randomly divided into four groups (n = 5) for remineralization evaluation. Fresh CMCS/ACP, CMCS/AuNPs/ACP, and CMCS/AuNPs/NaF were applied on the surface of acid-etched tooth enamel samples with small brushes, respectively. DW was used as the negative control group. The nanohybrids were smeared daily and then immersed in 10 mL of artificial saliva (PH 6.5–7.0; 1.5 mM CaCl_2_, 0.9 mM K_2_HPO_4_, 130 mM KCl, 1 mM NaN_3_, and 20 mM HEPES buffer), and replaced with fresh artificial saliva daily incubated at 37°C for 1 week. At the end of the 1-week treatment, the enamel samples were rinsed several times with DW and then dried in a vacuum drying oven at 25°C. Scanning electron microscopy (SEM, VEGA3, TESCAN, CzechRepublic) was used to observe the surface morphology of the samples. X-ray diffraction (XRD) spectra were recorded by X-ray diffractometer (Rigaku Ultima IV, Japan) to identify crystal structures. The samples were examined between 10° and 60° (2*θ*) at a scanning rate of 5° (2*θ*) per minute equipped by Cu Kα radiation (λ = 0.15418).

### 2.7 Cytotoxicity assay and biocompatibility *in vivo* assessment

Cell Counting Kit-8 (CCK-8) assays were performed on the human oral keratinocytes (HOKs) to evaluate the cytotoxicity. HOKs purchased from Wuhan Pricella Life Technology Co., Ltd. Use generations four through six for the following tests. After routine resuscitation, HOKs were cultured in an incubator (WIGGENS, Germany) at 37°C, 5% CO_2_, and 95% relative humidity in a completed α-MEM medium (Solarbio, China) containing 10% fetal bovine serum (FBS, Solarbio, China) and 1% penicillin/streptomycin (Biological Industries, Israel). HOKs were inoculated into 96-well microdrop plates with a cell density of 5×10^3^ cells per well and incubated for 24 h. The medium was replaced with 100 µL fresh medium containing varying concentrations of nanohybrids ranging from 0.3 μg/mL to 2.5 μg/mL. After an additional 24-h incubation, the medium was replaced with 100 µL of α-MEM that contained 10% CCK-8 (Dojindo, Japan) per well and then incubated at 37°C for 2 h. The absorbance was measured at 450 nm using a fully automated enzyme labeling detector (Bio Tek, United States). For each sample, the absorbance is the average of three parallel measurements. Furthermore, the cytotoxicity of CMCS/AuNPs/ACP nanohybrids was qualitatively analyzed using a calcein-AM/propidium iodide (PI) double staining kit.

The animal experimental protocol was approved by the Ethics Committee of Qingdao Stomatological Hospital (2022KQYX024). All animals were treated humanely during the whole experiment. Four-week-old Sprague-Dawley rats were selected for organ toxicity assessment, and the CMCS/AuNPs/ACP nanohybrids were fed to the rats by gavage every day. Group A: CMCS/AuNPs/ACP nanohybrids at a concentration of 2.1 μg/mL; Group B: no treatment. After 1 week, all rats were euthanized. The hearts, livers, spleens, lungs, and kidneys of rats in each group were collected and fixed in 10% formalin solution, and embedded in paraffin overnight. Then the paraffin tissue sections were dehydrated in graded alcohol, soaked in xylene three times for 10 min, and stained with hematoxylin and eosin (H&E) to observe the morphology under the optical microscope (BX43, OLYMPUS, Japan).

### 2.8 Statistical analysis

All data are presented as mean ± standard deviation (SD) of the independent experiment. One-way analysis of variance (ANOVA) was used for multiple comparisons. All statistical analyses in this paper were performed on at least three parallel experiments without special declaration: *p < 0.05, **p < 0.01, ***p < 0.001.

## 3 Results and discussion

### 3.1 Characterization

**SCHEME 1:**
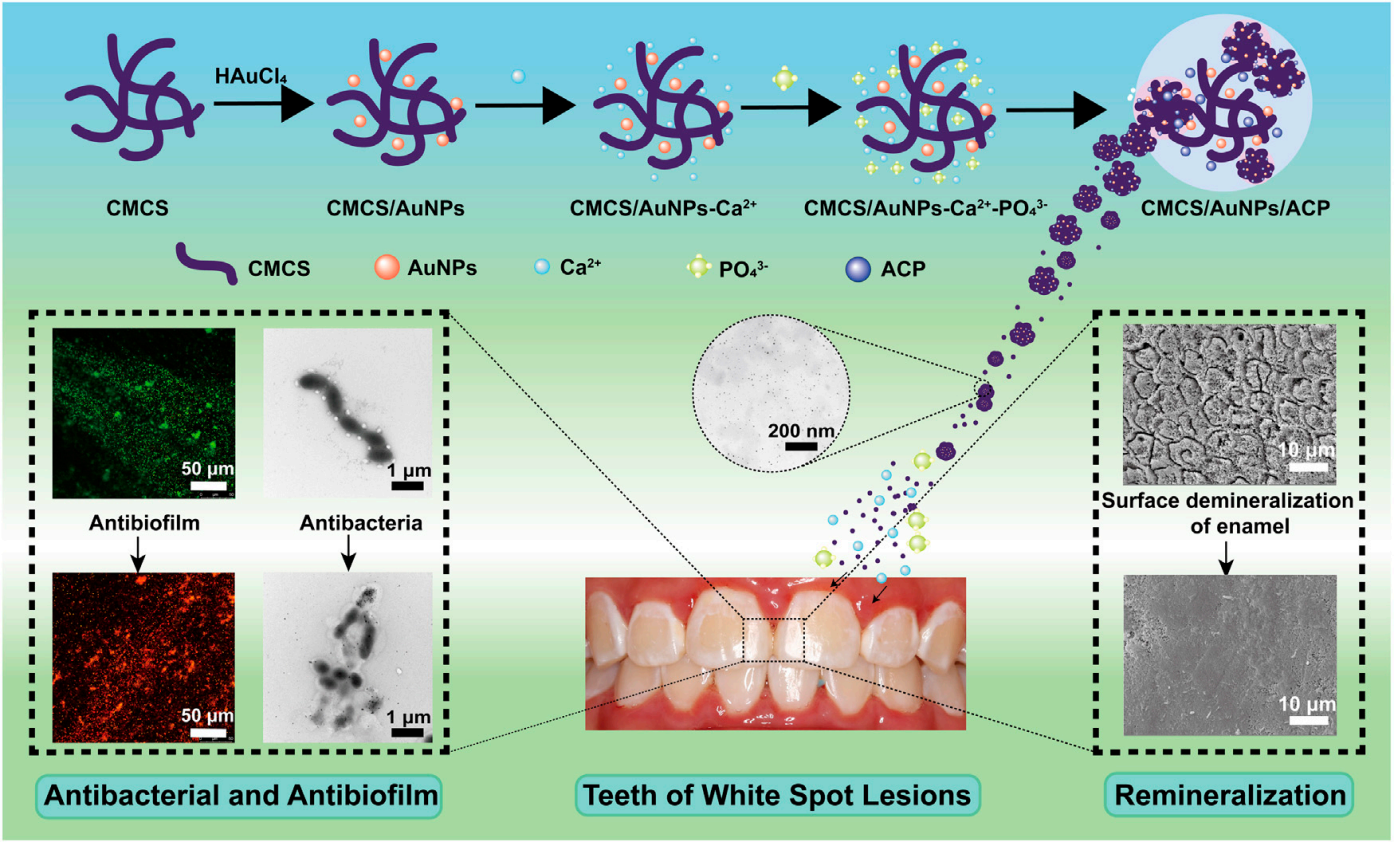
Schematic representation of the preparation of CMCS/AuNPs/ACP nanohybrids and their antimicrobial and remineralization properties.

In order to control dental plaque and achieve enamel remineralization, CMCS/AuNPs/ACP nanohybrids were constructed. CMCS *in situ* reduced and stabilized AuNPs possess enhanced antimicrobial activity, which could damage the cariogenic biofilm. Meanwhile, large amounts of Ca^2+^ and PO_4_^3-^ ions released by ACP provided the necessary elements for remineralization, especially combined with the ability of CMCS/AuNPs to induce the formation of HAp, which ultimately achieved the remineralization of enamel. The schematic representation of the preparation of CMCS/AuNPs/ACP nanohybrids and their antimicrobial and remineralization properties is shown in Scheme 1.

**FIGURE 1:**
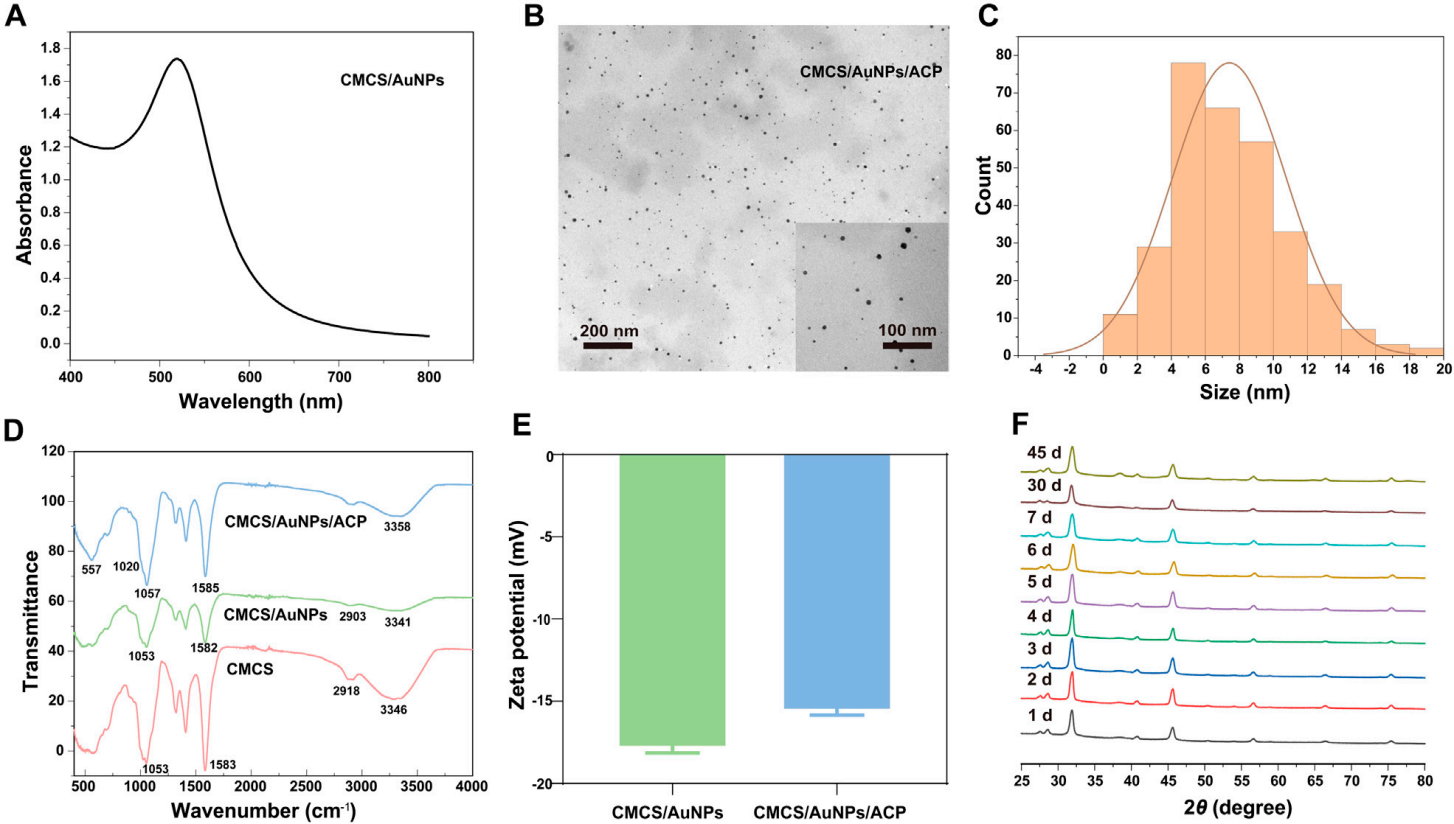
Characterization of nanohybrids. **(A)** UV-Vis absorption spectra of CMCS/AuNPs. **(B)** TEM image and particle size distribution of CMCS/AuNPs/ACP. **(C)** The particle size distribution of AuNPs. **(D)** ATR-FTIR spectra of CMCS, CMCS/AuNPs, and CMCS/AuNPs/ACP. **(E)** Zeta potential of CMCS/AuNPs and CMCS/AuNPs/ACP. **(F)** The XRD image of CMCS/AuNPs/ACP from day 1 to day 7, day 30 and day 45.

CMCS not only acts as a reducing agent but also as a stabilizer to synthesize stabilized AuNPs. The size and yield of AuNPs depend on the concentrations of CMCS, HAuCl_4_, and NaOH as well as the reaction time and temperature (Sun et al., 2019). The synthesized AuNPs were confirmed by UV-Vis spectroscopy, which showed a characteristic peak at 519 nm (Sun et al., 2019) (Figure 1A). TEM images showed that AuNPs in CMCS/AuNPs/ACP hybrid materials were uniformly dispersed spherical particles without aggregation, and the AuNPs size was 7.4 ± 3.3 nm (Figures 1B, C). CMCS contains amino functional groups and the broadband at 3,346 cm^-1^ in the FTIR spectra of CMCS/AuNPs corresponds to the -NH_2_ group and -OH group. In the preparation of AuNPs under alkaline conditions, the - OH and - NH_2_ groups of the CMCS play an important role in the reduction of Au(III) to Au (0) (Selvakannan et al., 2003; Newman and Blanchard, 2006). The FTIR spectra of CMCS/AuNPs showed a blue shift of the C=O and N-H bending peaks (1,582 cm^-1^) of the former as compared to that of CMCS (Figure 1D) (Ibrahim et al., 2020), which may be attributed to the formation of coordination bonds between the AuNPs and the nitrogen/oxygen atoms in the molecular chain of CMCS (Huang et al., 2007). The amino and carboxyl groups in CMCS play an important role in stabilizing AuNPs (Selvakannan et al., 2003; Huang et al., 2007). FTIR spectra of CMCS/AuNPs/ACP detected vibrational peaks at 557 cm^-1^ (*ν*_4_P-O) (Rey et al., 2017; Simeonov et al., 2019), indicating the formation of CMCS/AuNPs/ACP nanohybrids (Figure 1D). The zeta potentials of CMCS/AuNPs and CMCS/AuNPs/ACP were −17.8 ± 0.5 mV and −15.6 ± 0.4 mV, respectively (Figure 1E). Through the XRD results of CMCS/AuNPs/ACP from day 1 to day 7, day 30 and day 45, we can see that the material composition does not change, and there is no characteristic peak of HAp (He et al., 2022). The above characterization fully demonstrated the successful preparation of CMCS/AuNPs/ACP nanohybrids.

### 3.2 *In vitro* antibacterial performance

**FIGURE 2:**
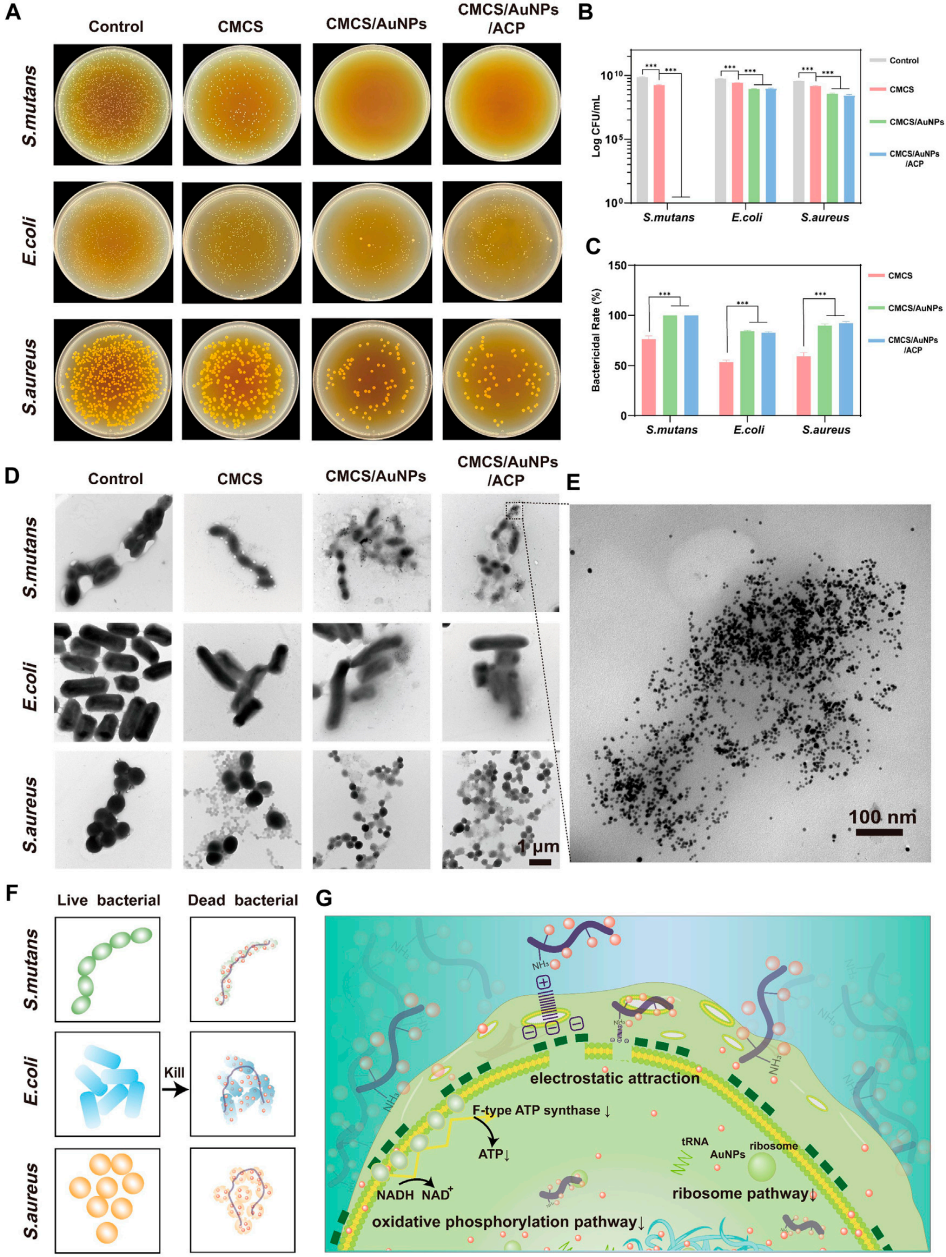
Antimicrobial properties of nanohybrids. **(A)** Representative photographs (10^8^-fold dilution), **(B)** the log value of the CFU counts and **(C)** quantitative results of survival bacteria colonies on agar plates after incubating the bacterial suspension with CMCS, CMCS/AuNPs and CMCS/AuNPs/ACP nanohybrids (n = 3). **(D)** TEM images of *S. mutans*, *E. coli*, and *S. aureus* after incubation with nanohybrids for 24 h (Scale bar: 1 µm). **(E)** TEM images of *S. mutans* after incubation with CMCS/AuNPs/ACP for 24 h (Scale bar: 100 nm). **(F, G)** Schematic representation of antibacterial mechanisms of the CMCS/AuNPs/ACP nanohybrids.

The antibacterial activity of the nanohybrids against oral bacteria *S. mutans* (Gram-positive), *S. aureus* (Gram-positive), and *E. coli* (Gram-negative) is assessed by the spread plate method. The antimicrobial efficacy of CMCS, CMCS/AuNPs and CMCS/AuNPs/ACP was quantified by determining the number of colony-forming units (CFU) in agar plates. As shown in Figures 2A–C, the bactericidal rate of *S. mutans* in the CMCS-treated group was 76%, in contrast, no bacterial colonies were observed in the CMCS/AuNPs/ACP-treated groups, demonstrating nearly 100% bacterial inhibition rates of the nanohybrids against *S. mutans*. Similarly, the number of colonies was significantly reduced after co-culture of the nanohybrids with *E. coli* and *S. aureus*, and the bactericidal rates were 83% and 92%, which were 30% and 33% higher than those of the CMCS-treated groups, respectively. All these results indicated that AuNPs effectively enhanced the antimicrobial effect of the nanohybrids, and CMCS/AuNPs/ACP showed a stronger antimicrobial effect against Gram-positive bacteria. In contrast, there was no statistically significant difference in the bactericidal rates of the CMCS/AuNPs-treated group and the CMCS/AuNPs/ACP-treated group against the three bacteria, which proved that ACP had no effect on the antimicrobial effect of the nanohybrids.

After co-culturing with the nanohybrids for 24 h, the microscopic morphology of bacteria was observed by TEM (Figures 2D, E). The bacteria of the control group retained their original morphology, the cells were essentially of the same size and had a complete and smooth surface. In contrast, the bacteria were slightly reduced, and the cell membrane image was attenuated in the CMCS group due to the weak antibacterial activity. However, after exposure to CMCS/AuNPs/ACP nanohybrids, AuNPs accumulated around the bacteria, and the bacterial cell membranes were severely disrupted. In addition, bacteria were significantly smaller in volume compared to AuNPs exposure, possibly due to bacterial shirinkage following cell membrane destruction.

CMCS is a water-soluble chitosan derivative with biodegradability. Based on the available research reports, we hypothesized that CMCS-stabilized nanohybrids would not be degraded in the slightly acidic environment of caries (pH∼5) (Wei et al., 2021). The antimicrobial effect of CMCS/AuNPs/ACP on bacteria may be mediated by the electrostatic attraction of CMCS, which carries the -NH_3_^+^ cation, to the surface of negatively charged bacterial cells, which disrupts the bacterial cell membrane, inhibits the production of ATP or inhibits tRNA binding to ribosomal subunits (Figures 2F,G) (Fei et al., 2001; Cui et al., 2012; Bindhu and Umadevi, 2014). Small AuNPs can penetrate the bacterial interior, causing further damage, inactivation, and ultimately cell death. The smaller the particle, the larger the specific surface area, the greater the ability to penetrate the cell membrane, and the better the bactericidal effect (Bindhu and Umadevi, 2014). The antimicrobial effect may depend on factors such as the concentration of gold nanoparticles, particle size, and type of combined antimicrobial agent (Hernández-Sierra et al., 2008; Ahmady et al., 2019; Yang et al., 2019).

**FIGURE 3:**
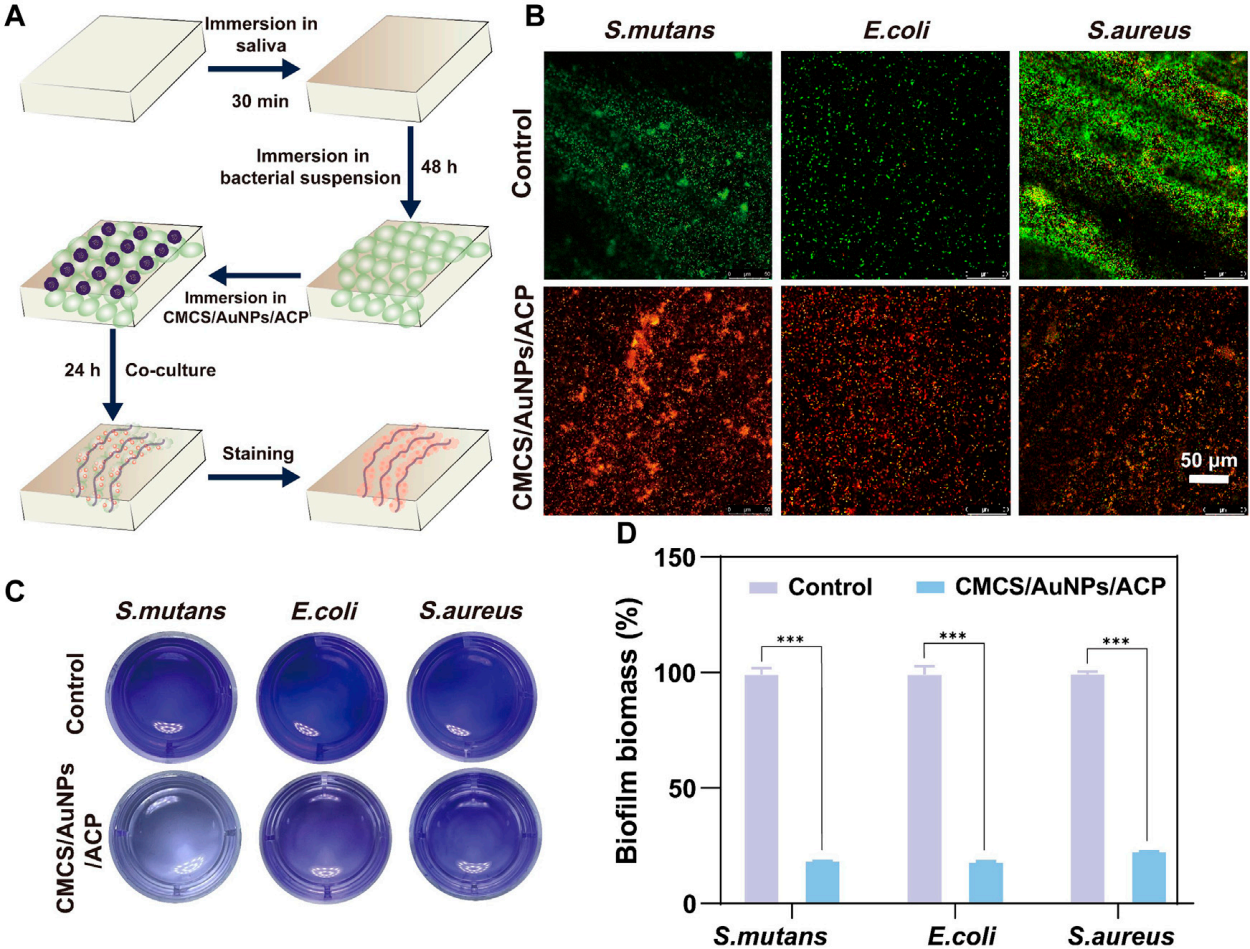
Antibiofilm properties of nanohybrids. **(A)** Schematic diagram of the experimental process of antibiofilm on tooth enamel surfaces. **(B)** Live/dead fluorescence staining images of *S. mutans*, *E. coli*, and *S. aureus* on enamel samples after PBS and CMCS/AuNPs/ACP nanohybrids treatment (Green, live bacterial; red, dead bacteria). **(C)** Crystalline violet stained images and **(D)** percentage of viable bacteria after 24 h incubation with PBS and CMCS/AuNPs/ACP nanohybrids.

The anti-biofilm activity is evaluated by Live/Dead fluorescence staining and the results after treatment with CMCS/AuNPs/ACP nanohybrids are displayed in Figure 3B. The control group showed strong green fluorescence. In contrast, nearly no green spots are observed from the CMCS/AuNPs/ACP-treated enamel surface, indicating excellent anti-biofilm activity. Crystal violet staining method is used to quantify the anti-biofilm effect of the CMCS/AuNPs/ACP nanohybrids. As exhibited in Figures 3C, D, the biomass of *S. mutans*, *E. coli*, and *S. aureus* biofilms in the CMCS/AuNPs/ACP nanohybrids group was reduced by approximately 81%, 83%, and 77%, respectively, which was significantly reduced compared to the control group. The results strongly prove that CMCS/AuNPs/ACP nanohybrids can cause effective damage to bacterial biofilms.

### 3.3 Enamel remineralization *in vitro*

**FIGURE 4:**
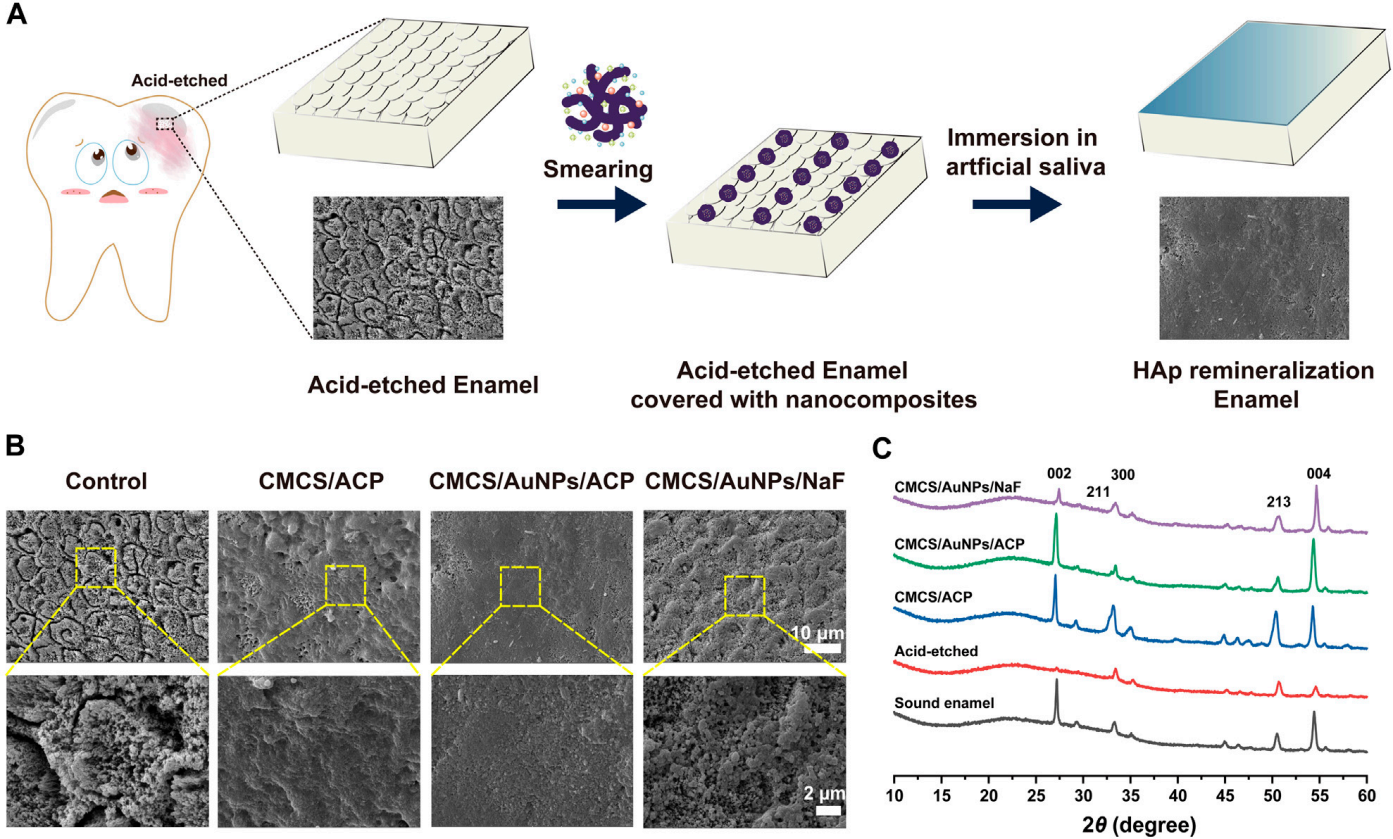
Evaluation of the effect of the CMCS/AuNPs/ACP nanohybrids on enamel remineralization *in vitro*. **(A)** Schematic diagram of the experimental process of remineralization of demineralized tooth enamel surfaces. **(B)** Scanning electron microscope images of demineralized enamel surfaces treated with CMCS/ACP, CMCS/AuNPs/ACP, and CMCS/AuNPs/NaF, respectively, and immersed in artificial saliva for 1 week. **(C)** XRD patterns of sound enamel, untreated demineralized enamel, CMCS/ACP-treated, CMCS/AuNPs/ACP-treated, and CMCS/AuNPs/NaF-treated demineralized enamel after 1 week of incubation in artificial saliva.

Once the hard tissues of the teeth are damaged, they cannot repair spontaneously. Therefore, it is necessary to inhibit tooth demineralization and promote tooth mineralization. The effect of CMCS/AuNPs/ACP on enamel remineralization was investigated using an *in vitro* model to evaluate its potential application for arresting WSLs (Figure 4). Firstly, the remineralization ability was analyzed by observing the morphology of tooth enamel. The results showed that enamel exposed the shape of “fish scales” with prismatic structures after being eroded with 37% phosphoric acid. The surface of CMCS/ACP-treated demineralized enamel formed a partially remineralized layer, with prisms still exposed, and the surface was rough and porous, indicating that CMCS/ACP could not recover the enamel tissue well. Meanwhile, the enamel treated with CMCS/AuNPs/NaF (NaF, 2000 ppm) had flaky and random mineral deposits on the surface, and the “fish scale” morphology was still visible. By comparison, a dense structure was observed on the enamel surface after CMCS/AuNPs/ACP treatment, which demonstrated the enamel remineralization ability of CMCS/AuNPs/ACP nanohybrids.

The nanoparticles formed from these remineralized layers are likely to be newly formed HAp nanocrystalline, and carboxyl and hydroxyl groups have been reported to be the most effective apatite formation sites (Toworfe et al., 2006), and amino acid-stabilized AuNPs induce the formation of hydroxyapatite and effectively inhibit crystal size growth (Sari et al., 2022). We therefore hypothesize that the CMCS-stabilized AuNPs may induce the crystallization process involving ion-exchange reactions by providing a surface to bind Ca^2+^ and PO_4_^3-^ in the ACP, thereby facilitating the crystallization of the mimetic HAp, CMCS/AuNPs combined with ACP nanoparticles can penetrate well into the prisms and form HAp nanocrystalline of uniform size (Sari et al., 2022). Whereas after NaF replaced ACP, although new fluorapatite particles can be formed, the particle homogeneity and densification are significantly lower than those of the ACP group. In CMCS/AuNPs/ACP nanohybrids, not only ACP can provide Ca^2+^ and PO_4_^3-^, but also CMCS/AuNPs can provide a surface to induce the crystallization process, which increases the penetration of Ca^2+^ and PO_4_^3-^ into the prismatic spaces and promotes the formation of dense remineralized layers.

In addition, the crystal structure of the surface of different enamel samples was analyzed by XRD. The results showed that the diffraction peaks of (002) (211) (300) (213), and (004) at 2*θ* = 27.0, 32.8, 33.2, 50.4, and 54.3° were all characteristic peaks of HAp (Figure 4C), which appeared on enamel, and the diffraction patterns of the three groups of remineralization were similar to those of the normal enamel, which indicated that they had a similar crystal structure. On the surface of the acid-etched enamel samples, the reflection peaks of (002) (300) (213), and (004) were obviously weakened or disappeared. The distinctive diffraction peaks were evidently reinstated in the deminleralized enamel samples treated with CMCS/ACP and CMCS/AuNPs/ACP, indicating that the newly generated crystalline HAp on the samples, which led to the remineralization, whereas the diffraction peaks of the CMCS/ACP-treated group were narrow, indicating that the crystalline grains were large, which was consistent with the SEM results. The crystallinity of the CMCS/AuNPs/NaF-treated demineralized enamel samples was significantly lower than that of the CMCS/ACP-treated group and the CMCS/AuNPs/ACP-treated group, which indicated that the remineralization effect of NaF was weaker than that of ACP.

### 3.4 *In vitro* and *in vivo* biocompatibility of CMCS/AuNPs/ACP

**FIGURE 5:**
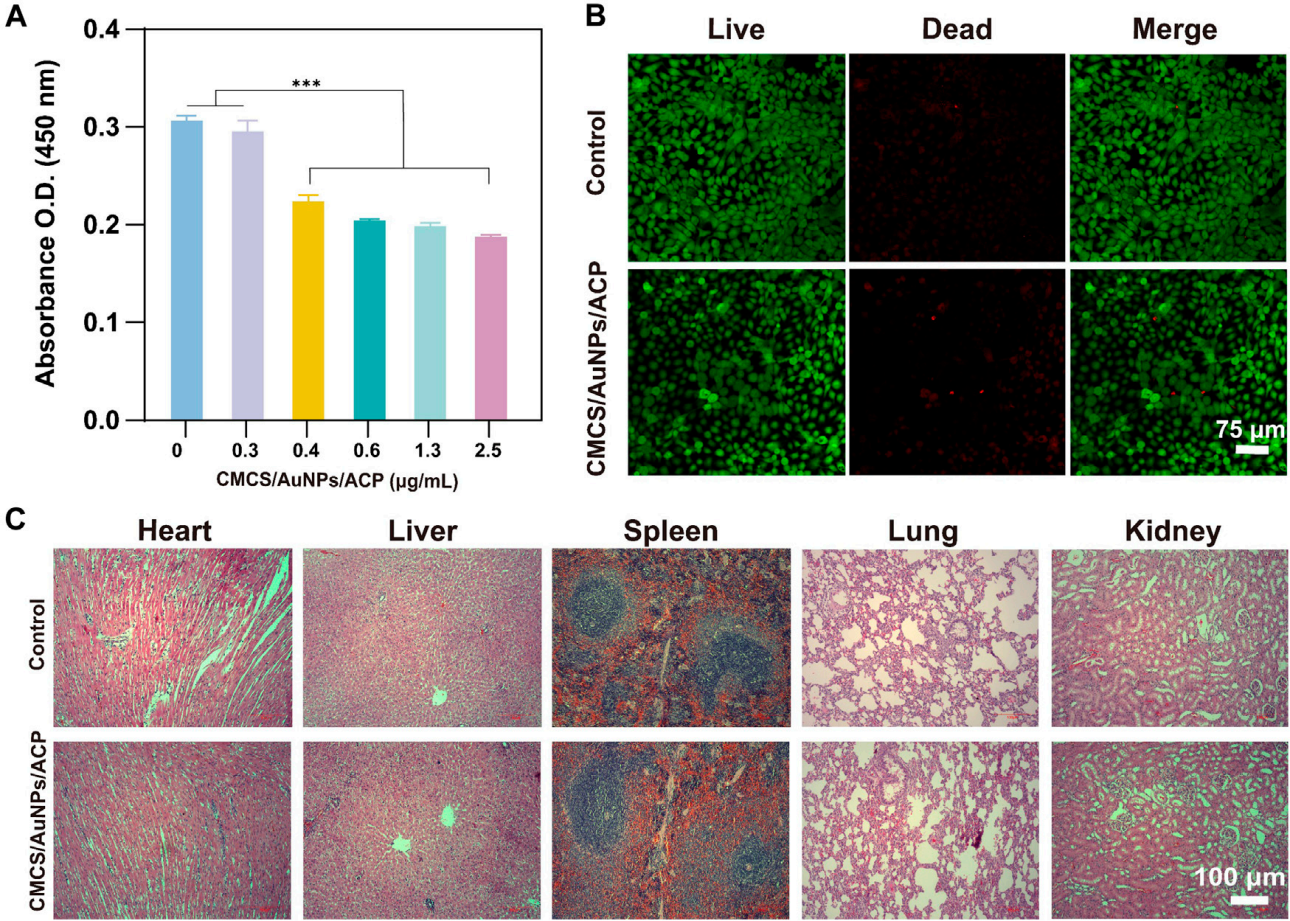
*In vitro* and *in vivo* biocompatibility tests of CMCS/AuNPs/ACP nanohybrids. **(A)** The cytocompatibility of CMCS/AuNPs/ACP after incubation with HOKs for 24 h **(B)** CLSM images of HOKs treatment with CMCS/AuNPs/ACP for 24 h **(C)** H&E-stained images of the major organs of SD rats fed with CMCS/AuNPs/ACP nanohybrids on 7^th^ day.

The ideal remineralization solution should have good biocompatibility as it will be used in the mouth. Therefore, in order to fully evaluate the biocompatibility of our CMCS/AuNPs/ACP, we performed both *in vitro* (cytocompatibility) and *in vivo* tests. After different doses of CMCS were co-cultured with HOKs for 24 h, the effects of CMCS/AuNPs/ACP on HOKs viability were determined by CCK-8 assay and living/dead cell viability assay. As the concentration of CMCS/AuNPs/ACP increased, the number of cells showed a gradual decrease, indicating that the toxicity of nanohybrids was concentration-dependent, and the nanohybrids at a concentration of 0.3 μg/mL were almost non-toxic compared with the blank control group. Therefore, the proper addition of CMCS/AuNPs/ACP nanohybrids has little effect on cell viability. The fluorescence analysis showed that almost all cells had a strong green signal and normal polygon morphology after 24 h of CMCS/AuNPs/ACP action (Figure 5B), indicating that the nanohybrids had no effect on biological growth in the biological applications.

To perform *in vivo* biocompatibility tests, SD rats were fed nanohybrids by gavage for 7 days. Subsequently, major organs (heart, liver, spleen, lungs and kidneys) were observed by H&E staining. CMCS/AuNPs/ACP did not appear to cause any tissue defects compared with the control group (Figure 5C). These results indicate that CMCS/AuNPs/ACP has excellent biocompatibility with potential for *in vivo* medical applications.

## 4 Conclusion

CMCS/AuNPs/ACP nanohybrids have excellent antimicrobial and remineralization properties, the bactericidal rate of *S. mutans* is close to 100% and can destroy the attachment of bacterial biofilm, and a dense HAp remineralization layer may be formed on the surface of demineralized enamel through the combination of Ca^2+^ and PO_4_^3-^ supplied by ACP, under the guidance of CMCS/AuNPs, which can provide a clinical treatment of enamel WSLs with new ideas and possibilities.

## Data Availability

The raw data supporting the conclusion of this article will be made available by the authors, without undue reservation.
